# Worm Grunting, Fiddling, and Charming—Humans Unknowingly Mimic a Predator to Harvest Bait

**DOI:** 10.1371/journal.pone.0003472

**Published:** 2008-10-22

**Authors:** Kenneth C. Catania

**Affiliations:** Department of Biological Sciences, Vanderbilt University, Nashville, Tennessee, United States of America; Georgia State University, United States of America

## Abstract

**Background:**

For generations many families in and around Florida's Apalachicola National Forest have supported themselves by collecting the large endemic earthworms (*Diplocardia mississippiensis*). This is accomplished by vibrating a wooden stake driven into the soil, a practice called “worm grunting”. In response to the vibrations, worms emerge to the surface where thousands can be gathered in a few hours. Why do these earthworms suddenly exit their burrows in response to vibrations, exposing themselves to predation?

**Principal Findings:**

Here it is shown that a population of eastern American moles (*Scalopus aquaticus*) inhabits the area where worms are collected and that earthworms have a pronounced escape response from moles consisting of rapidly exiting their burrows to flee across the soil surface. Recordings of vibrations generated by bait collectors and moles suggest that “worm grunters” unknowingly mimic digging moles. An alternative possibility, that worms interpret vibrations as rain and surface to avoid drowning is not supported.

**Conclusions:**

Previous investigations have revealed that both wood turtles and herring gulls vibrate the ground to elicit earthworm escapes, indicating that a range of predators may exploit the predator-prey relationship between earthworms and moles. In addition to revealing a novel escape response that may be widespread among soil fauna, the results show that humans have played the role of “rare predators” in exploiting the consequences of a sensory arms race.

## Introduction

In a number of parts of the southeastern United States, families have handed down traditional knowledge for collecting earthworms by vibrating the ground. This technique is variously called worm grunting, fiddling, snoring, and charming (hereafter called worm grunting after the yearly “Worm Grunting Festival” in Sopchoppy, Florida). The strategy consists of a range of methods by which man-made vibrations are communicated to the soil, either by using hand tools or occasionally power equipment (more recently chain saws but historically a model T ford might be used). As a result of these vibrations, earthworms exit their burrows and can be easily collected. Although commonly used to collect fishing bait on a small scale, this technique seems to have reached its greatest level of development in Florida's Apalachicola National Forest, where an entire bait industry developed in the 60's and 70's with thousands of people grunting for worms for supplemental income or as the major means of supporting their families. Worm grunting, and the astounding results by which literally thousands of large worms can be collected in only a few hours, attracted national news coverage in 1972 [1], [2]. Earthworm collection in the Apalachicola National Forest was subsequently scrutinized by the Forest Service and regulated following concerns that the large endemic earthworms might be over-harvested (primarily *Diplocardia mississippiensis* Smith [3]). A yearly permit is now required for harvesting worms and powered methods of generating vibrations are prohibited.

The ubiquitous local knowledge of this technique, and its use to support a small industry, are a testament to the strength of the earthworm's behavioral response to vibrations. It is clearly a dangerous behavior for worms, and seems counterintuitive given that–irrespective of human bait collectors - terrestrial earthworm predators rooting through soil could produce ground vibrations, and therefore the opposite response (moving deeper into the soil) might be predicted. In fact, both wood turtles (*Clemmys insculpta* LeConte) and herring gulls (*Larus argentatus* Pontoppidan) have been reported to vibrate the ground in order to capture emerging earthworms [4], [5]. Even in the absence of a specific surface predator, worms emerging onto the soil surface in daylight expose themselves to opportunistic predation and desiccation. This raises an obvious question: why do earthworms surface in response to vibrations?

Charles Darwin discussed clues to this behavioral response in his work on earthworms [6]. He stated, “It has often been said that if the ground is beaten or otherwise made to tremble, worms believe that they are pursued by a mole and leave their burrows.” And later “Nevertheless, worms do not invariably leave their burrows when the ground is made to tremble, as I know from having beaten it with a spade, but perhaps it was beaten too violently”. The possibility that worms interpret vibrations as a digging mole has also been suggested in some popular accounts of worm grunting, though Darwin's account is sometimes quoted [7] and thus could be the origin of the suggestion.

It has also been suggested that earthworms may respond to vibrations caused by rainfall and exit their burrows to avoid drowning. In much of North America, it is common to see earthworms on the soil or pavement after prolonged rain, adding support to the latter suggestion. It also seems possible that vibrations may be a novel and aversive stimulus that could elicit an escape response without corresponding to a naturally occurring threat.

The goal of the present investigation was to test the first hypothesis, as recounted by Darwin [6] - that some worms have evolved an escape response to ground-borne vibrations to avoid foraging moles. A number of questions come to mind in considering this possibility. For example, given that the large earthworms native to the Apalachicola National Forest have a particularly strong response to vibrations–do moles inhabit the area? What is the potential impact of mole predation on earthworms–i.e. how many earthworms can moles eat? Do earthworms exit their tunnels during a rainstorm? Are worms in danger of drowning in wet soil? How do the vibrations created by a worm grunter compare to those of a digging mole? How do earthworms respond to a digging mole?

These questions are addressed by a series of studies and observations that begin with a description of worm grunting and subsequent earthworm responses, followed by an examination of mole tunneling and distribution in the Apalachicola National Forest, and finally an investigation of how earthworms respond to rain, saturated soil, burrowing moles, and recordings of a digging mole. The results show that the earthworms from the National Forest (*Diplocardia*) respond to moles by rapidly exiting the soil to flee across the surface and suggest that humans have unknowingly learned to mimic the vibrations caused by a digging mole to collect bait. Preliminary observations suggest a range of other species of earthworms may also escape from moles by detecting vibrations and exiting to the soil surface, where moles do not forage.

## Results

### Worm Grunting

**Figure 1:**
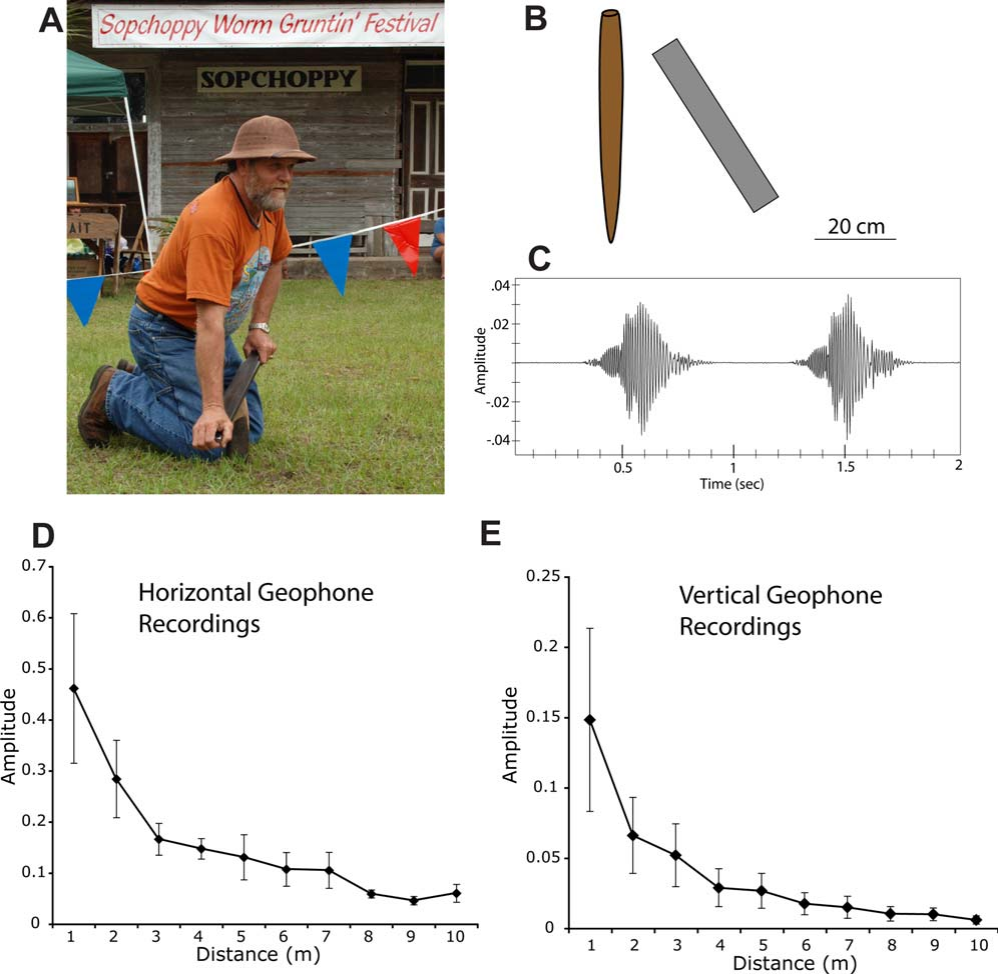
Worm grunting demonstrated and described. A. Gary Revell, a professional bait collector, demonstrates worm grunting at the Annual Worm Grunting Festival in Sopchoppy Florida on April 12 of 2008. See movie S1 for an example of worm grunting during bait collection. B. This technique requires a wooden stake and a flat piece of iron. The stake is driven into the ground and the iron is rubbed across the surface as in plate A. C. A two second recording of worm grunting vibrations made with a vertically oriented geophone at a distance of 5 meters (supplementary audio file S1). Two strokes were made during the two seconds, each lasting roughly 400 milliseconds. D, E. The relative amplitude of vibrations recorded at successive 1-meter intervals from the vibrating stake. Units are arbitrary but all recording parameters were constant throughout the study. The intensity of the horizontally recorded vibrations (n = 5) was stronger than vertically recorded vibrations (n = 3). Bars are standard error of the mean.

Observation and description of this technique (under National Forest Service earthworm harvesting permit number WAK40) were made possible by the generous help of Gary and Audrey Revell. They have been collecting earthworms in the Apalachicola National Forest using this method for much of their lives and they provide yearly demonstrations at the annual Sopchoppy Worm Grunting Festival (Figure 1). The method requires two tools: a wooden stob, or stake and a rooping iron, or long piece of steel, such as an automobile leafspring. The wooden stakes are of variable size and shape, but generally about 4–8 cm in diameter and 30–60 cm in length, tapered to a crude point on one end so that they can be driven into the ground. The irons are also of variable size and shape, but usually at least 40 cm in length, 4–8 cm in width and of variable thickness with a flat area that can be rubbed over the wooden stake (e.g. Figure 1B).

After the stake is driven into the ground, vibrations are produced by rubbing the flat part of the iron lengthwise across the stake (see movie S1). When properly performed, friction between the two materials causes low frequency stick-slip vibrations that are propagated through the soil while simultaneously producing the audible “grunting” sound that gives this technique its name. Each stroke and the corresponding vibrations typically last less than a second and this is repeated many times at each collecting site.

Geophone recordings (Figure 1) allowed for several basic parameters of the vibrations to be determined. Both vertical and horizontal geophone recordings were taken at increasingly distant, 1-meter intervals while vibrations were generated at 5 different sites. The peak energy content of the vibrations was concentrated at approximately 80 hz (see later section for details). The stroke and corresponding vibrations lasted for an average of 610 milliseconds (n = 20). Movie S1 and audio file S1 provide a demonstration of the technique and the sound file for vibrations recorded from the vertically oriented geophone at 5 meters distance. The relative magnitude of the vibrations fell off steeply with distance (Figure 1D, E) but there was considerable variation in the intensity of propagated vibrations at different sites, as would be expected given heterogeneous soil structures throughout the forest. The horizontal component of the vibrations was most intense.

### Worm Responses to Grunting

**Figure 2:**
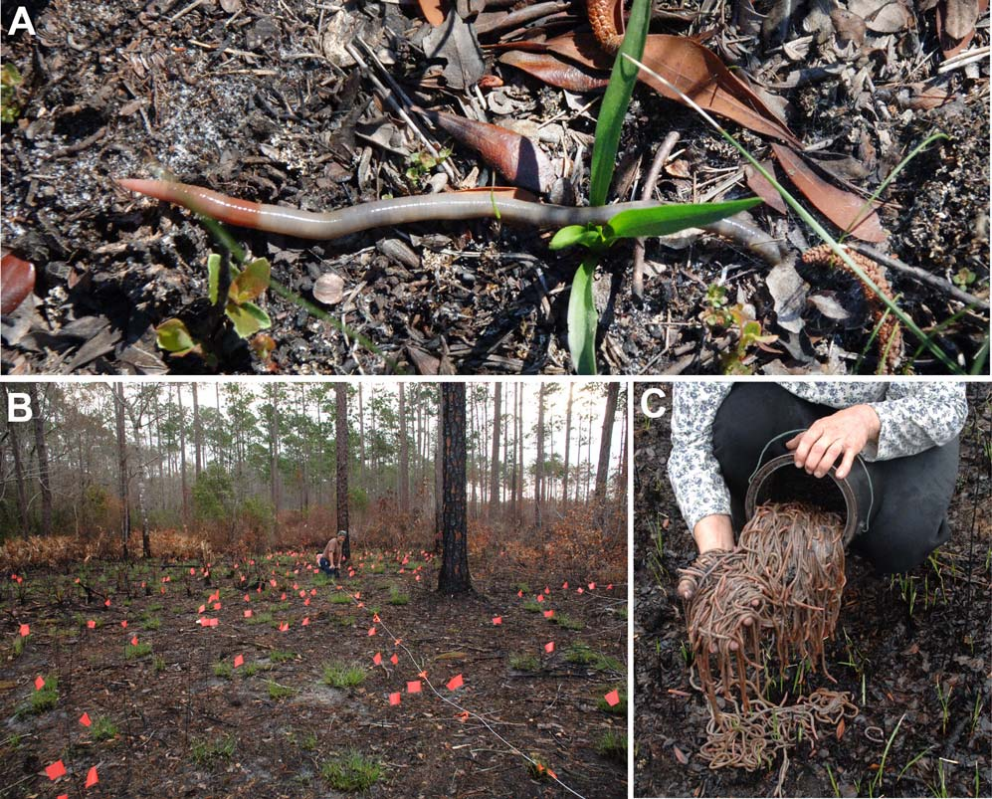
Observing Gary and Audrey Revell at work. A. In response to the vibrations, earthworms exit their burrows. B. By marking the worms as they emerge, their numbers and distribution were determined. Note that Gary Revell is in the center of the image, and earthworms have emerged for up to 12 meters from his location (flags). C. Audrey Revell shows the results of just 2 stake placements (roughly 500 worms).

**Figure 3:**
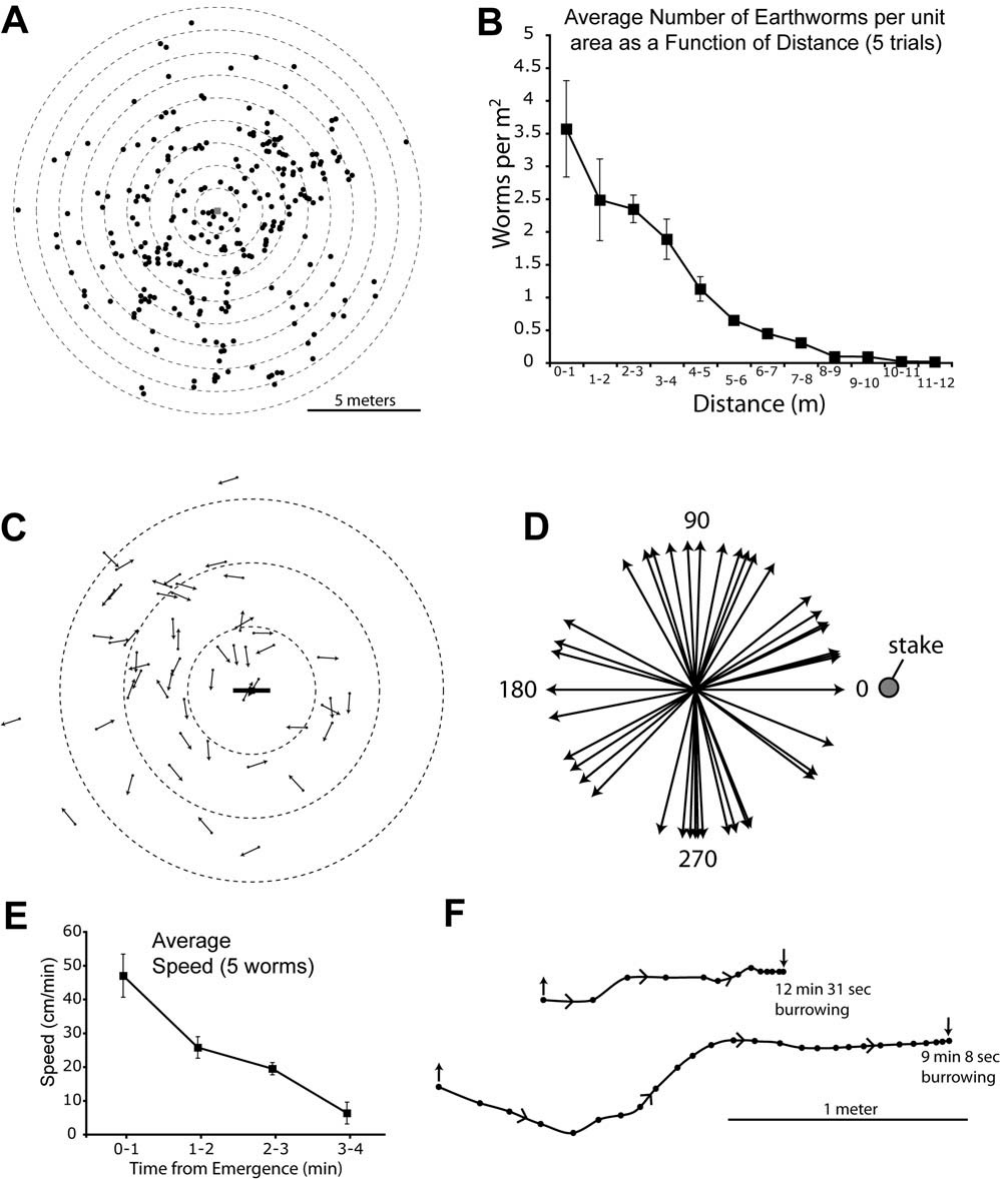
Responses of earthworms to worm grunting vibrations. A. The distribution of 262 earthworms (black dots) that emerged from a single stake placement (center). Dotted circles are incremental meter distances from the stake. B. The average number of earthworms that emerged per square meter at each distance for 5 trials. Few worms emerged beyond 10 meters from the stake. C. The location and direction of movement relative to the vibrating stake for 52 worms. Dotted circles have 1, 2, and 3 meter radii. D. The direction of movement of each worm (arrows) in relationship to the stake (circle). No significant direction preference was found (Rayleigh test; P = 0.261, Z = 1.35). E. The mean speed of 5 worms that emerged as a result of vibrations. Their speed progressively decreased over time. F. The paths of 2 worms showing their point of emergence (up arrow) their position every 30 seconds (dots) and the relative location at which they burrowed back into the ground (down arrow), with burrowing time indicated.

In response to the vibrations made at a number of sites within the Apalachicola National Forest, hundreds of large earthworms rapidly emerged from the ground for a distance of up to 12 meters from the location of the vibrated stake (Figure 2). Upon emergence, each worm began to travel across the soil surface. Measurement of movement for 18 worms at an unknown time from initial emergence gave an average speed of approximately 30 cm/minute. The direction of movement relative to the vibrating stake appeared to be random, and this was subsequently confirmed in a different set of trials (see below). At 5 different locations the position of each earthworm was marked with a flag as it was collected (Figure 2B) and the total number of earthworms that emerged at each 1-meter interval from the stake was determined. Figure 3A shows the complete distribution of 262 collected earthworms from a single site from one trial. The density of emerging worms consistently decreased with increasing distance (Figure 3B). Few worms (10 total in 5 trials) emerged beyond 10 meters from the stake.

To determine whether earthworms traveled in a particular direction relative to the vibrating stake, the location of emergence and direction of movement was documented for 52 worms (Figure 3C). These directions were subsequently translated into angles relative to the stake (Figure 3D) and no significant directional preference was found (Rayleigh test; P = 0.261, Z = 1.35).

Under most circumstances, the goal of worm grunting is to rapidly collect the emerging earthworms and then move to another, adjacent site for additional collections. But what do the earthworms do if not collected? In the course of the present investigation, many earthworms were observed after their emergence to address this question. Worms that were not collected began to burrow back into the ground after traveling some distance. In general, worms emerged from their tunnels traveling at the highest speed, subsequently reduce their speed over time for several minutes, and then began to probe the soil for a favorable area to burrow. The path that worms took was fairly straight, though obstacles (vegetation) often caused a change in direction. This sequence was documented in detail for 5 earthworms (Figure 3E), and the entire sequence is illustrated for two worms in Figure 3F.

Although precise times were recorded for only 5 burrowing earthworms, it was clear that returning to the soil could take considerable time. The mean time for 5 worms to completely disappear into the soil was 49 minutes. However this time was dependent on soil conditions, and some worms took less than 10 minutes (e.g. Figure 3F). Worms that emerged from the soil at mid-day began to burrow the soonest, and took the longest time to re-enter the dry, hot soil, whereas worms that emerged at dawn on moist ground traveled the farthest and returned to the soil in the shortest time. It seemed obvious that worms were in danger of desiccation and sensed the relative heat and moisture content of the soil surface. A few worms that emerged during the heat of the day (31°C) in unshaded regions were unable to return to the soil before dying. In several cases, worms were attacked by ants, snakes, lizards, or beetles, before returning to the soil.

To summarize, in response to the vibrations caused by a bait collector, hundreds of earthworms rapidly emerged from their burrows for a distance of roughly 10 meters in all directions. The worms began to travel over the soil surface in random directions at maximal speed, decreased their speed over time until, and after approximately 4–15 minutes (depending on temperature and moisture) began the process of returning to the soil. The burrowing process lasted from approximately 10 minutes in moist areas to over an hour in areas with drier soil.

### Moles in the Apalachicola National Forest

**Figure 4:**
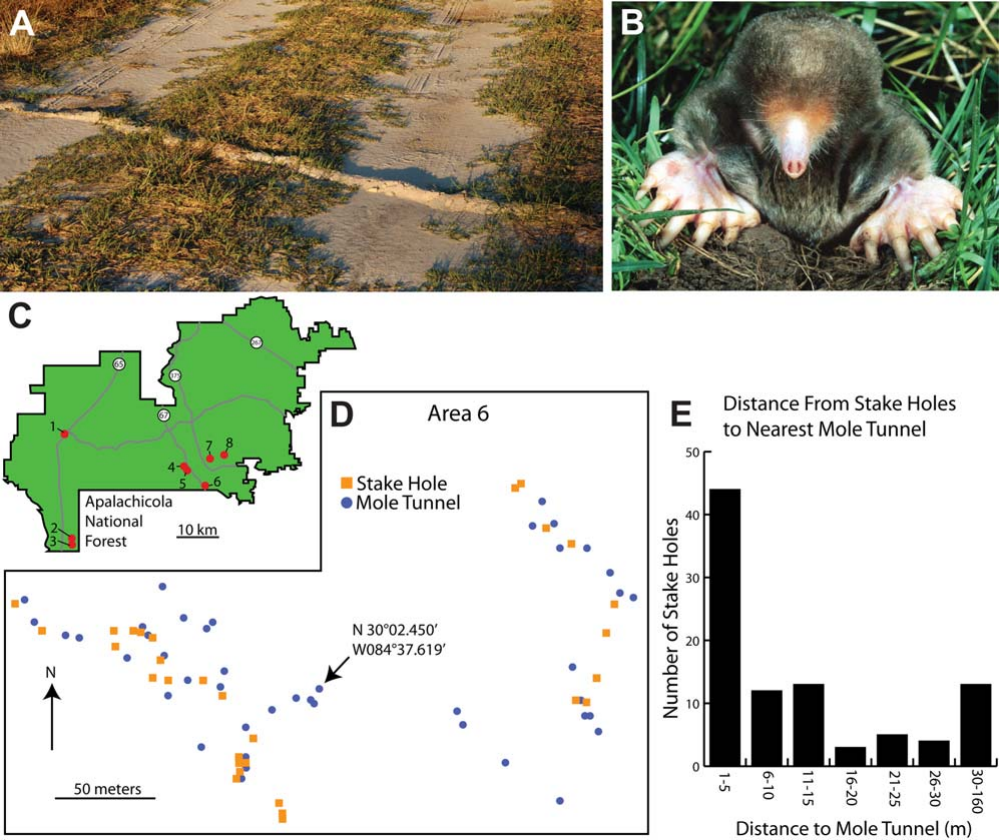
Eastern American moles (*Scalopus aquaticus*) in the Apalachicola National Forest. A. One of 39 noted road incursions by moles on designated forest roads. B. An eastern American mole (*Scalopus aquaticus*) showing the large forelimbs used to excavate tunnels. C. A schematic of the Apalachicola National Forest showing major roads (gray) and the 8 bait collection sites (red circles) examined for mole tunnels. D. The relative location of stake holes from bait collectors (orange squares) and mole tunnels (blue circles) for site 6. In every location examined, bait collection areas overlapped with mole tunnels. E. Histogram showing the number of stake holes at given distances from mole tunnels, compiled from all 8 sites. Ninety four stake holes and 204 mole tunnels were identified.

The eastern American mole (*Scalopus aquaticus* Linnaeus) lives in much of the eastern United States and is the only mole found in the Florida panhandle [8]. Although its range includes the Apalachicola National Forest, there have been no studies of its local abundance within the forest's borders. Thus initial observations were aimed at simply searching for mole tunnels within the forest and accessing soil and vegetation conditions. But from the outset of this study it was apparent that large populations of *S. aquaticus* occupy the area. This was evident from the multiple and recent incursions of moles into many officially designated and maintained forest roads (Figure 4A). Thirty-nine mole tunnels were noted that were dug across or into 11 different roads. Tunnels in this tally were at least 100 meters from one another, suggesting the presence of separate moles [9]–[11, and personal observation].

More definitive evidence of an overlap between the endemic earthworm populations and eastern moles became clear while accompanying the Revells to various collections sites. In every location, mole tunnels were identified within a relatively short distance of areas where worms were collected. To examine this systematically, stake holes from previous baiters were identified in 8 different locations separated by at least 1 km (Figure 4C). At the same time, nearby mole tunnels that could be located were marked at each location (Figure 4D). Ninety four stake holes and 204 mole tunnels were noted in total from the 8 areas. For 44 stake holes, representing 47% of the total, a mole tunnel was identified within a 5 meter distance. The average distance to the nearest mole tunnel for all of the stake holes was just under 20 meters. The furthest distance from a stake to a mole-tunnel was 160 meters. Clearly, moles live in the areas where bait is collected by worm grunting.

Activity within numerous mole tunnels was confirmed by placing wooden dowels vertically into the tunnels. These were pushed aside when the mole traversed its tunnel, thus indicating recent passage of the animal without disturbing the burrow system. Using this technique, three moles were captured by hand as they traveled through their tunnels.

Previous studies in our laboratory suggest that captive eastern moles can eat the equivalent of their body weight in commercially available nightcrawlers every day. To access this potential in regard to *Diplocardia* earthworms, a single mole from the Apalachicola National Forest was fed a diet of endemic *Diplocardia* purchased from the Revell's bait shop. After one week of acclimation, this 42 g mole continued to eat an average of 23 worms per day weighing an average of 42.4 g in total weight (as measured for a 10 day period). This intake represents just over 15 kg per year, though certainly fewer worms would be eaten in the wild (see discussion).

### Responses of Worms to Moles and Rain

**Figure 5:**
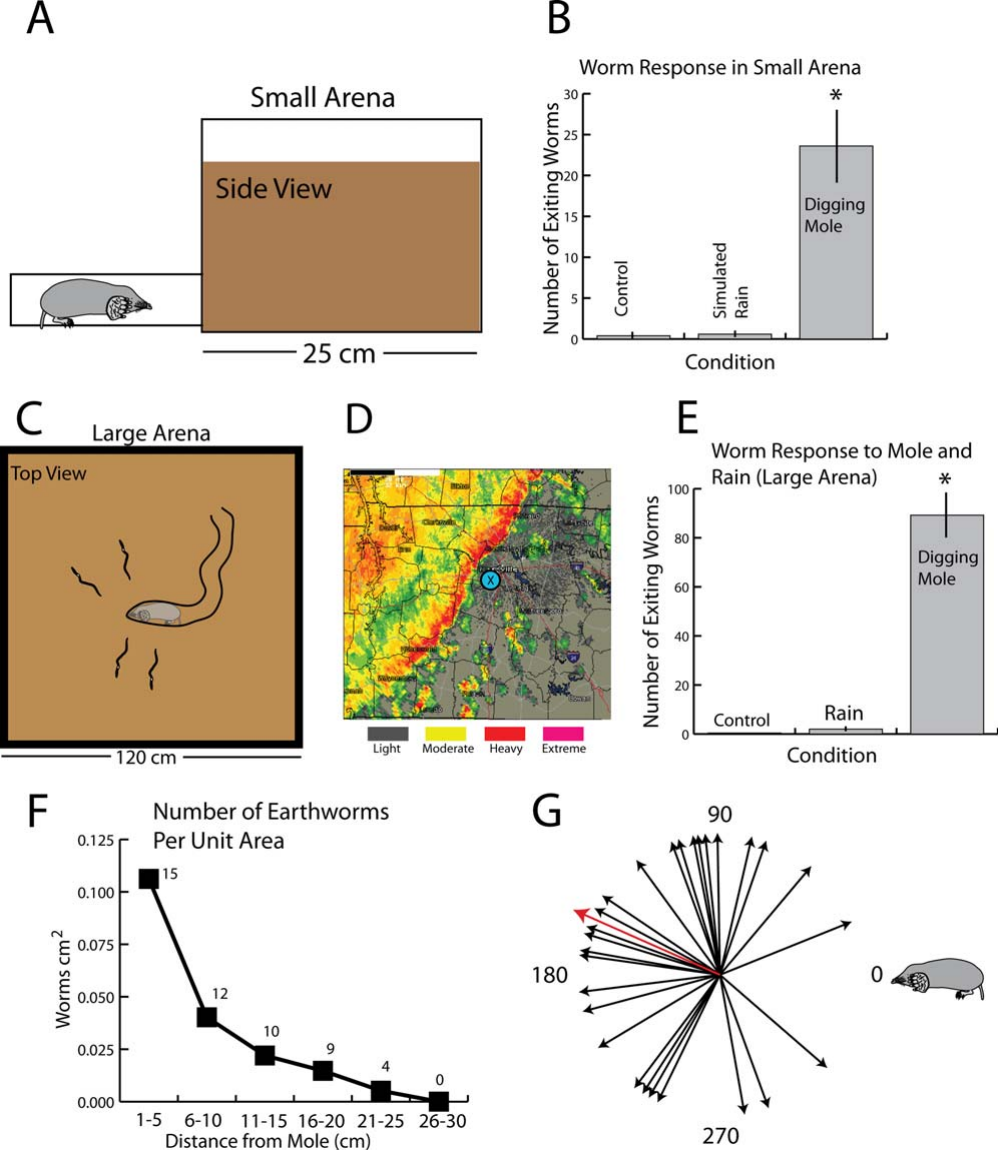
Summary of experiments examining earthworm responses. A. Preliminary tests were performed in a small arena (20 w×25 l×19 h cm) containing soil and 50 *Diplocardia* earthworms. The mole entered through a tube in the lower corner and observations were made for 1 hour. B. Histogram illustrating responses. In 5 trials, each lasting 1 hour, an average of 23.6 earthworms exited to the soil surface, usually shortly after the mole entered and disturbed the soil. Movie S2 shows the first part of one trial. The control period was a 1 hour interval prior to the test. Simulated rain consisted of a sprinkler system that provided a simulated downpour at a rate of 1 inch per minute as measured by a rain gage. * indicates significant difference between digging mole and other conditions (*F*_(2,12)_ = 26.44, *p*<0.0001). Bars are SEM. C. Schematic illustration (top view) of the large, outdoor arena (1.2×1.2 m filled to a depth of approximately 15 cm) used for a more natural setting. D. Weather radar showing the relative rainfall for a thunderstorm on April 1st, 2008, 00:31 hours, during which observations of *Diplocardia* responses were made. Observations were made during the first hour of rainfall for each trial (rainfall was continuous) and included periods of moderate and heavy rain. X marks the approximate location of the outdoor arena. E. Responses of earthworms in the large arena to a digging mole for 1 hour (5 trials) and 1 hour of moderate to heavy rain (3 trials). See movies S3 and S4 for responses to digging moles. * indicates significant difference between digging mole and other conditions (*F*_(2,10)_ = 70.66, *p*<0.0001). Bars are SEM. F. The number of worms that emerged at different distances from the mole for 50 observations. Y-axis units represent worms per unit area as summed for the 50 trials (and thus are arbitrary). Numbers for each square represent the raw total of worms for each distance. G. Summary of the directional preference for movement of the escaping worms for 30 observations. The earthworms had a significant direction preference (Rayleigh test P = 0.034; Z = 3.33) with a mean vector of 156 degrees (mole at zero degrees) as indicated by the red arrow.

Having established that endemic moles and worms coexist in the Apalachicola National Forest, the next set of experiments was aimed at determining whether the earthworms respond to a digging mole. In the first, preliminary set of trials, a small (20×25×19 cm) container was used to house 50 worms and a mole was allowed to enter through a habit-trail tube in the lower corner (Figure 5A). The container was filled with soil to a depth of approximately 15 cm, and the earthworms were placed on the surface and allowed to burrow. After the earthworms had acclimated overnight, the bin was observed for 1 hour as a control period prior to each trial and any worms that had emerged before or during the 1 hour period were counted and removed. In five trials, 2 worms were removed from the soil surface during the control period. These were replaced with new worms that burrowed into the soil. The trials were then begun by introducing a mole through the tube and observing the results for 1 hour.

The earthworms exhibited a marked response with a short latency–specifically, many worms rapidly exited to the soil surface and attempted to exit the area, often by crawling over the container walls. A videotaped trial is included as movie S2. Earthworms seemed to have an escape response in the presence of moles. In this regard, it should be noted that eastern moles do not exit to the soil surface while foraging (see discussion), thus fleeing to the surface provides worms both immediate safety and the most efficient means for movement away from the predator for subsequent burrowing. In 5 trials, an average of 23.6 worms, or 47%, exited the soil within 1 hour (Figure 5B). In the different trials, the moles exhibited variable levels of activity, and each trial appeared to include relatively long periods of inactivity.

As a preliminary test for potential responses to rain and saturated soil, 50 worms were once again allowed to burrow into the soil for each of 5 boxes as described above. Each was then placed under a continuous sprinkler system that provided a simulated downpour at a rate of 1 inch per minute as measured by a rain gage. Each box was observed for 1 hour, and any worms that emerged were counted and removed. In 5 trials, 3 worms emerged from the soil in the containers during these trials (Figure 5B). As would be expected, by the end of the trials the soil was completely saturated and there was standing water on the soil surface of the containers. The soil was then removed from the containers and the earthworms were examined. In each case, the worms appeared healthy and had suffered no obvious deleterious effects.

Following these trials, 2 larger outdoor arenas measuring 1.2 m^2^ were constructed and filled with soil to a depth of approximately 15 cm to provide a more natural setting for the observations (Figure 5C). Three hundred *Diplocardia* were then placed within each arena and allowed to burrow. After the earthworms had acclimated overnight, the containers were observed for 1 hour as a control period prior to the trial and any worms that had emerged before or during the 1 hour period were counted, removed, and replaced with new worms. A mole was then placed on the soil surface, allowed to burrow, and the results were observed for one hour. These procedures were repeated for a total of 5 large-bin trials.

The moles dug tunnels in various directions at different intervals, and this behavior and the corresponding surface ridges appeared indistinguishable from behavior and tunnels that were observed in the field (see later section). In response to the digging mole, many earthworms exited the soil and traveled across the surface (movie S3). For the 5 trials, an average of 89 worms, or approximately 30%, exited to the surface. In these more natural trials, the potential utility of this response was more apparent, as the worms seemed clearly to be escaping from the digging mole. Many of the worms exited when the mole was quite close (5–10 cm) but some worms exited at a distance of 20 centimeters or more (e.g. movie S4). In contrast to the behavior resulting from worm grunting, many of the earthworms appeared to have a directional response and moved away from the mole.

To document distance and direction of emergence more carefully, additional trials were performed with the camera in the same plane as the soil surface. Fifty earthworm escapes were filmed in this manner, and the distribution of distances from the mole to the emerging worm are shown in figure 5F. The direction of travel for the earthworms was also measured relative to the position of the mole for 30 trials (Figure 5G). The worms were found to have a significant directional preference (Rayleigh test P = 0.034, Z = 3.33) with a mean vector of 156 degrees, approximating a path away from the mole (at zero degrees).

To examine potential responses to rain, the large arenas described above containing 300 earthworms each were observed during thunderstorms accompanied by moderate to heavy rainfall, for a total of 3 large-bin trials. The average rainfall for the 3 trials was ½ inch per hour as measured by a rain gage within the bin. The local weather radar for the period just prior to one of these trials (12:31 am, April 1st, 2008) is illustrated in figure 5D, with the approximate location of the large arena indicated. In the course of these three trials a total of 6 earthworms emerged to the soil surface. In each case, these few worms emerged after at least 25 minutes of steady rain. By the end of these trials, the soil was saturated and there was standing water on the soil surface (the arenas had no drainage holes). In the next 12–24 hours, depending on weather conditions, the soil was turned and the earthworms were examined and appeared healthy.

### Vibrations Caused by Moles

**Figure 6:**
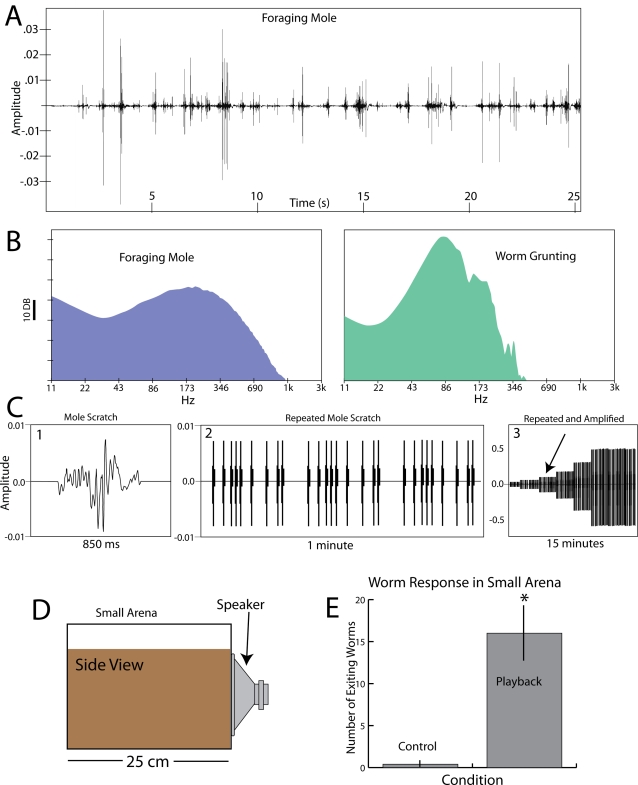
Vibrations caused by a foraging mole and earthworm responses. A. Vertical geophone recording of a wild, foraging mole in Davidson county TN, from a distance of approximately 15 cm (see supplementary audio file S2). B. Representative spectrums of a foraging mole (from the first 23 seconds of the recording above) and a worm grunter (from the segment in figure 1C). C. Recording of a single scratch from a foraging mole (1). This scratch was repeated multiple times (2) and then amplified over time (3) to simulate a digging mole. Arrow marks the point in the playback at which earthworms consistently emerged from the soil. D. Small arena used in playback experiments. E. Results of playback experiment. In 5 trials and average of 16 earthworms surfaced in response to the simulated mole. * indicates a significant difference (*t*(8) = −4.712, p<0.005, indicating significant difference between digging mole and control period (see methods).

Moles are powerful diggers that disturb the soil considerably as they use their forelimbs to extend tunnels and search for prey. Often, a mole digging in the wild is clearly audible to an observer standing several feet away (see movie S5). Sounds and corresponding ground vibrations are generated as the mole forcefully moves soil, scrapes its claws through the soil, and especially when networks of small roots (ubiquitous in most of their habitat) are broken. A number of geophone recordings were made as wild, foraging eastern moles extended their tunnels in Davidson County in Tennessee. A 25 second example of these vibrations recorded with a vertically oriented geophone from a distance of approximately 15 cm is shown in figure 6A (and see supplementary audio file S2). The peak amplitude of these vibrations was similar to the amplitude of vibrations caused by a worm grunter at a distance of approximately 6–10 meters (Figure 1). The frequency components (power spectrum) of a worm grunter and a digging mole are compared on a log scale in figure 6B. As might be expected, the worm grunter vibrations are more uniform, concentrated near 80 hz. The foraging mole produced a wider range of vibrations with the strongest peak near 200 hz.

To examine how worms responded to vibrations caused by a digging mole, a section of a recording representing a single scratch (Figure 6, C1) was copied into a new file and repeated at varied time intervals with silence between scratches (Figure 6, C2). This sound track was then amplified over time in an attempt to simulate an approaching mole for a 15 minute duration (Figure 6, C3). The entire sound track was then repeated 4 times, such that 1 hour included 4 simulated “mole approaches”. These stimuli were then played through a speaker into the soil in the small arena containing 50 earthworms as previously described (Figure 6D). In 5 trials, an average of 16 earthworms surfaced during the 1 hour time period (movie S6). In each case, the earthworms began to emerge during the 3^rd^ step of amplification. To obtain an approximate measure of the vibrations generated at this stage, a geophone was placed in the center of arena during playback. The amplitude of the vibrations was similar to those obtained from a worm grunter at a distance of 8–10 meters.

### Wild Moles

Although it was not possible to locate a wild mole actively extending its tunnel in the Apalachicola National Forest, such observations were possible in Davidson County Tennessee. This allowed for geophone recordings of naturally occurring foraging behavior, as previously described. It also provided a striking example of earthworm escape responses occurring under natural conditions. In the course of roughly one hour of videotaped observations, more than 60 earthworms exited the soil near the burrowing mole (see movie S5). The mole could literally be tracked across the soil surface by the trail of escaping worms. In addition, 3 insect larvae exited the soil and traveled rapidly across the surface.

## Discussion

The results of this investigation support the hypothesis that earthworms have a stereotyped escape response from foraging moles, and that bait collectors have unknowingly learned to mimic digging moles to flush worms. The escape response consists of rapidly exiting the soil, which prevents pursuit by the mole, and allows efficient movement away from the mole for subsequent burrowing at a more distant location.

The Apalachicola National Forest provided an ideal setting for this investigation for several reasons. First, there is a long history of bait collecting as a means of support for many families in and around the forest. This suggests that earthworms in the area have a particularly strong response to vibrations and begs the question of why they should surface, exposing themselves to a host of terrestrial predators. Second, these bait collection practices continue to this day, allowing for observation and study of a technique that has been handed down for generations. In this respect, I am indebted to Gary and Audrey Revell for their generosity in demonstrating how and where bait collection takes place, and for sharing their extensive knowledge of the forest ecosystems. Finally, the area is largely undeveloped and has both a native earthworm population and a native mole population, suggesting the “sensory arms race” between these moles and earthworms has a long evolutionary history. This is not trivial, given that human introduction of earthworms across continents [12], [13] has made such relationships difficult to access in many areas.

The results raise a number of questions for further discussion and study. For example, how widespread is this response among earthworm species and what other species might exhibit such escape behavior? How does this newly described response compare to other well-studied systems, such as echolocating bats and flying insects? What are the mechanisms and nervous system specializations that might account for the response? What predators may exploit the longstanding predator-prey interactions between moles and earthworms and what other invertebrates may respond in this manner? These and other questions are discussed below.

### *Diplocardia* Responses to Rain

The results of this investigation, including observations within the National Forest, suggest that worm grunting does not simulate rainfall. Evidence for this conclusion comes from the simulated rain experiments, during which few worms emerged, and the exposure of earthworms to thunderstorms with heavy rain, which produced similar results. In both cases, only a few worms exited the soil after a long latency (>15 minutes). In neither case did earthworms in saturated soil appear to be in distress. In fact, more long-term observation of *Diplocardia* earthworms housed in outdoor arenas suggested the threat of desiccation was greater than that of drowning in a sudden downpour. Worms that remained in completely saturated soil for over 24 hours appeared in good health. In addition, no emerging worms were observed during one rainstorm within the Apalachicola National Forest (personal observation). Finally, the behavior of *Diplocardia* during worm grunting does not seem an appropriate adaptation to avoiding drowning. This impression comes from watching earthworms emerge in full daylight, during warm weather, onto hot, dry substrate. It seems unlikely that other strong sensory cues about moisture content in the environment would be over-ridden by vibrations, or that rapid emergence and movement in a random direction (Figure 3D) would be adaptive at the onset of rain (e.g. *Diplocardia* do not move uphill in response to vibrations). By contrast, the short latency of the response and rapid movement (for an earthworm) over the soil surface are appropriate for escaping a subterranean predator that does not surface to pursue prey (personal observation, and see [14], [15]). In this respect, the response is reminiscent of flying fish that can exit the water to travel briefly through the air where aquatic predators cannot follow [16]. For both prey items, the foray into the hostile environment is short-lived, but allows re-entry to the predator's realm at a more distant location.

Why then are earthworms observed on the surface after heavy rains? Perhaps the most obvious explanation is that a number of species of earthworms in different habitats are, in fact, potentially in danger of drowning after prolonged rainfall. For example, Chuang and Chen [17] recently examined oxygen consumption and surfacing behavior in 2 species of earthworms and found that one species (*Pontoscolex corethrurus*) had a lower rate of oxygen consumption and never emerged from the soil after heavy rain. The other (*Amynthas gracilis*) had a higher rate of oxygen consumption and did surface after heavy rain. Thus some earthworms may be more sensitive to oxygen depletion in saturated soil [18] than others. But it is important to note that under conditions simulating heavy rain with saturated soil, the average time until *A. gracilis* emergenced was 10 hours, and the earthworms usually emerge after nightfall. This is consistent with the general observation that earthworms are often observed on the surface the morning after a heavy rain, but does not suggest these earthworms have a short-latency response to the onset of rain that might be cued by vibrations.

### The Rare Enemy Effect

Perhaps the most interesting facet of these results is that humans are unknowingly cast in the role of the “rare enemy” that exploits a prey's adaptations to a more common threat. Dawkins [19] outlined this scenario, suggesting that a predator with a comparatively small impact on prey relative to more common predators may develop and maintain a strategy that exploits the prey's behavior - and by extension its nervous system [see also 20 for exploitive mimicry]. This has been well documented for painted redstarts (*Myioborus pictus*) that use high contrast plumage and tail fanning to elicit insect flight while foraging [21]. These flush-pursuit predators are thought to activate the hard-wired escape circuitry of insects [22]–[25] and may even direct the prey into the most sensitive part of their visual field for efficient pursuit. Evolution of this strategy depends on the predominance of gleaning predators, for which escape by flight remains the best insect defense [21].

Remarkably, humans are not the only ones to flush earthworms using vibrations. Tinbergen [4] noted that herring gulls exhibit a foot-paddling behavior, which flushes worms from the ground in Europe. Moreover, he suggested the earthworm's innate response to vibrations was to escape moles: “*What I have seen in other gulls has*, *however*, *convinced me that paddling has two functions*. *One is the bringing up of earthworms*, *which seem to have an innate reaction to the quivering of the soil which is of value*, *enabling them to escape their arch enemy*, *the mole*.” He suggests the other reason was to flush and expose small animals in muddy pools of water, where foot paddling is often observed. This presumably more common practice for gulls suggests the origins of the behavior, which might easily be transferred to the terrestrial setting where it could be subsequently reinforced through individual experience, selection over generations, or both.

Kaufmann documented a second example in wood turtles, which also stomp the ground to flush earthworms [5], [26]. On over 200 occasions wood turtles were observed to stomp the ground while foraging, and this behavior often elicited emergence of earthworms that were pursued and eaten. Subsequent investigation revealed that others had independently observed the same behavior in wood turtles and the earthworm response [27]. Kaufmann was aware of bait collection techniques in the American southeast and specifically described the turtle's behavior as a form of worm grunting [28]. Like Tinbergen, Kaufmann attributed the earthworm's response to an escape behavior from moles.

Apparently, the idea that earthworms respond to vibrations to avoid foraging moles has been considered for some time, but never formally tested. It may be that most biologists wondering about earthworm behavior have read Darwin's work on the subject [6] and noted his comments on the matter. However both Darwin and Tinbergen [4] make reference to unpublished personal communications from others. This suggests that a number of naturalists have chanced upon a digging mole and noted escaping earthworms, as was observed in the present investigation. This in turn suggests such escape responses may be widespread for different earthworms responding to moles.

The apparently widespread responses of earthworms to moles, and the ability of predators to exploit these responses, depend on the predominant selective pressure exerted by foraging moles. What is the potential impact of moles on earthworms? Investigations of stomach contents of wild caught European moles (*Talpa europaea*) suggest they eat 60 g of food per day, with earthworms composing a large proportion of the diet [29], [11]. This represents over 20 kg per year, more than half of which is usually earthworms [11]. Studies of the eastern mole (*Scalopus aquaticus*) suggest they may consume similar quantities of invertebrates, with earthworms making up a large proportion of the diet [30], [31]. Our own experience with captive eastern moles, which we feed commercially available nightcrawlers (*Lumbricus*), indicates they may easily consume their body weight in worms each day. This was measured and confirmed for a single mole from the Apalachicola National Forest fed exclusively on *Diplocardia* earthworms collected by baiters. The 42 g mole consumed an average of 42 g of *Diplocardia* (23 per day) over a 10 day period (after 1 week of acclimation). This likely represents more than would be eaten in the wild. Even so, half of this amount would be 7 kg of earthworms per year, or roughly 3–4 thousand adult *Diplocardia* (6–8 times the number shown in figure 2C). Clearly moles represent an important potential predator of earthworms.

The interaction between moles and earthworms is reminiscent of the sensory arms race between bats and flying insects [32] but is far less obvious due to the subterranean nature of the species involved (it is also difficult to observe because moles, like earthworms, have their own predators and are themselves very sensitive to vibrations). Bats are also small mammals that can have a strong impact on invertebrate populations. Although echolocation has provided a means for bats to exploit the night skies and the vast resource of flying insects, it also provides an obligatory and strong cue signaling insects of their approach. Many diverse insects have developed bat-detecting ears and take evasive maneuvers in response to ultrasound. A number of moths exhibit a 2-tiered response, first changing course to fly away from the bat in response to low amplitude ultrasound, and then diving to the ground (and acoustic crypsis) in response to high amplitude ultrasound [32], [33].

### Worm Charming and Cues Detected by Earthworms

As is the case for bats, a mole digging a surface run in search of prey provides an inherent and potentially strong set of cues to prey as it approaches. Vibrations are an obvious component of these stimuli (Figure 6A), and this was the focus of the present investigation in large part to explain the efficacy of worm grunting. However, another potential cue was noted - that of local compression of the soil by the forelimbs during the power-stroke of digging. This lower frequency component was not obvious in geophone recordings, but could be imitated by briefly compressing the soil by hand. As was the case for vibrations, this stimulus also elicited escape responses from earthworms. This cue was not carefully investigated in the present study, in part for lack of a mechanism for producing controlled stimuli of sufficient force. Yet it seems probable that escaping earthworms detect both vibrations (as illustrated by worm-grunting) and localized compression of the soil when escaping from a mole - the latter indicating a mole is particularly close. The combination of these two cues might elicit a more pronounced escape response than either presented alone. Interestingly, worm “charming” as it is called in the United Kingdom, appears to rely on the latter stimulus. Worm charming does not occur on a commercial scale to support a bait industry, but there is an annual “World Worm Charming Championship” held each year in Willaston (near Nantwich in the UK) and an International Festival of Worm Charming, held in Blackawton, Devon. The main technique is to drive a pitchfork into the ground, and rock it back and forth. This compresses the soil for a short distance around the pitchfork, and elicits escapes response from earthworms. Unlike the 80 hz vibrations produced during worm grunting that carry many meters, worm charming with a pitchfork appears to carry less than a meter, and thus has less dramatic results. Yet it is remarkable that two potential cues exist that signal an approaching mole, and two different methods have been developed on different continents to exploit these different cues (the present results suggest the name might be changed from “charming” to terrorizing - for as Tinbergen put it, these cues signal the approach of the worm's arch enemy).

### Some Remaining Questions

The results raise a number of questions from the perspectives of ecology to neuroethology. For example, it would be of interest to investigate how widespread these escape responses may be among the soil fauna, and what other predators might exploit such responses. It may also be that a large proportion of earthworms in the Apalachicola National Forest can escape mole predation by detecting their approach, requiring moles to depend on other invertebrates [e.g. 34]. Moles in general are exquisitely sensitive to touch [35], and it would be of interest to examine whether moles have developed counterstrategies. For example a mole that interposed itself between the soil surface and an earthworm could detect its relatively large burrow and trap it. What happens on the coldest winter days, when worms may be inactive but moles are active and in particular need of prey? It may be that relocating territories in response to vibrations is essential for *Diplocardia* during warm weather, so that they are not vulnerable to predation during times of reduced activity. Finally, the nervous system of earthworms in the genus *Lumbricus* is well known for the giant fibers that mediate the rapid withdrawal response [36], [37]. Much has been learned about the electrophysiology of neurons and neuronal networks from such giant fiber systems, but it is often difficult to expand these physiological investigations to a natural setting. *Diplocaria* might provide such an opportunity.

## Materials and Methods

### Position Plots

The positions of mole tunnels and bait collection areas were marked with a Garmin hand-held Colorado 400t WAAS-enabled GPS unit with an accuracy of 3–5 meters (95% typical). Waypoints were downloaded into a Macintosh computer and imported into Google Earth. Distances between waypoints (Figure 4C) were plotted using the ruler function. Maps of waypoints (Figure 4D) were constructed by importing points into MacGPS Pro version 7.6, converting the plotted points to a Jpeg file, placing the file into Adobe Illustrator CS3 version 13, and then reconstructing the plots using symbols in Illustrator format. To plot earthworm positions in the field (Figure 3A), two Sonin 10300 Multi-Measure ultrasonic measuring units were used with receivers. The two receivers were placed several meters apart and measurements from each (previously marked) earthworm location were made to the nearest centimeter, one measurement to each receiver. These measures provided a unique position plot (for one side of the paired receivers) for each marked earthworm. The distance between the receivers was used to plot 2 (scaled) position markers representing the receivers in an Adobe Illustrator document. For each marked receiver point, the circle command was then used to create a circle with radius equal to the (scaled) distance from each receiver to each earthworm mark. The intersection of the 2 circles (each centered on the receivers location) represented the location of each marked earthworm, and these data are shown in figure 3A. To measure the shorter distances illustrated in Figure 3C, a tape measure was used in conjunction with a Strait-Line Model 120 laser level with degree marks to measure the distance and angle of the earthworm locations relative to the stake. The angle of the earthworm's path in relationship to the stake was measured with a segment of a folding wooden ruler, and then traced into a notebook. These angles were later scanned and placed in Adobe Illustrator, measured to the nearest degree, and illustrated in Figure 3C. The angles traveled relative to the stake were used to compose the schematic in figure 3D (see below for statistics). For distance measurements in large arena trials, a Cannon XL1 digital video camera was positioned in the same plane as the soil surface. Videotapes of a reference scale were made in the same focal plane as the trials. Earthworm escapes from a foraging mole were then recorded, imported into Imovie version 6.0.3 using a Sony DVMC converter box, and converted to Quicktime movies. Selected frames were exported from each trial and opened in Photoshop CS3 version 10. The track of each earthworm and the location of the mole based on soil movements were then marked on the digital image while reviewing the video segment. The mole's location was estimated as the central 4 cm of the soil disturbance at the time of earthworm escape–based on the consistent size of the mole tunnels. This file was then placed in Adobe Illustrator where distances and angle of movement relative to the mole were measured.

### Animal Collections

Earthworm collection from the Apalachicola National Forest was carried out under permit number WAK40. Moles from the Apalachicola National Forest were collected under state permit WX08126 and U.S. Department of Agriculture special use permit APA5098. Moles from Davidson County Tennessee were collected under state permit number 1868. Moles were collected by observing the deflection of wooden dowels as the mole traveled through its tunnel system, blocking the mole's passage with hand trowels, and then removing the mole by hand. *Diplocardia* used in the mole-earthworm interactions were purchased from the Revells' bait shop. Note that *Diplocardia* are not farmed and techniques for maintaining them long-term in an artificial natural setting have not been established. They are often maintained for bait in wood chips, but in this case they do not exhibit natural behaviors. To ensure healthy and active subjects, freshly collected specimens provided by the Revells were used for these investigations by arranging weekly deliveries. All procedures were approved by the Vanderbilt Institutional Animal Care and Use Committee and are in accordance with the National Institutes of Health guidelines for the care and use of animals in research.

### Geophone Recordings

Geophone recordings were made with Oyo Geospace geophones (Houston, TX) using a dedicated vertically or horizontally oriented model containing a GEO 11D transducer with a 4.5 Hz resonance frequency. The geophone output was through a coaxial cable that was connected to the audio input of a laptop without prior amplification or filtering. All signals were recorded on a Macintosh G4 computer using Audacity software version 1.2.6a with audio input set at 50%. Spectral analysis was performed in Audacity using the Fast Fourier Transform and plotted for log frequency (Figure 6B).

### Statistics

Directionality of earthworm movement was assessed using the Rayleigh test and *p* values were calculated using Oriana (Kovach Computing Services, Isle of Anglesey, Wales, UK) and were considered significant at *p*<0.05. For the small arena trials comparing the control period, simulated rain, and a digging mole (Figure 5B) data were analyzed using a one-way ANOVA as an omnibus test for a main effect of condition. This was followed by post-hoc *t*-tests. The same procedure was used to compare the control period, rain, and a digging mole in the large outdoor arena (Figure 5E). A *t*-test was used to compare the control period to playbacks of a digging mole (Figure 6E).

## Supporting Information

Audio File S1 Geophone recordings of worm grunting, as illustrated in figure 1C.
(0.02 MB MOV)

Audio File S2 This sound file plays amplified geophone recordings of a foraging mole as illustrated (without amplification) in figure 7A. It demonstrates some of the vibrations generated by moles as they forage.
(0.35 MB MOV)

Movie S1 Gary and Audrey Revell demonstrate worm grunting to collect bait in the Apalachicola National Forest in Florida's panhandle. The Revell's are professional bait collectors and make their living by collecting the large earthworms native to the area. These worms (Diplocardia mississippiensis) respond to vibrations by rapidly exiting their underground burrows. The vibrations are created by first pounding a wooden stake (called a “stob”) into the ground, and then rubbing the top of the stake with a flat piece of metal (a “rooping iron”). This is repeated in different areas until thousands of worms have been collected.
(8.61 MB MOV)

Movie S2 This video shows a preliminary test for earthworm responses to a burrowing mole. The container filled with dirt holds 50 Diplocardia earthworms. A mole is then introduced to the arena. As the mole digs, the earthworms exit to the surface and attempt to leave the area (video is sped-up).
(7.96 MB MOV)

Movie S3 In this video a mole burrows in the large arena filled with soil and containing 300 Diplocardia earthworms. This shows a more natural setting and illustrates the pronounced escape responses (sped up). Because burrowing moles generally remain below ground while hunting worms, a worm that exits to the surface is safe from the hungry mole. Moles generate vibrations and soil compressions as they dig, and the results of this study suggest that worm grunters are simulating moles.
(6.59 MB MOV)

Movie S4 In this video a mole burrows in the large arena filled with soil and containing 300 Diplocardia earthworms. This video is similar to video 3, showing a more natural setting and illustrating the pronounced escape responses (sped up), but in this case showing some of the responses at longer distances form the mole.
(4.44 MB MOV)

Movie S5 In this video a wild, foraging mole extends its tunnel in Davidson County, Tennessee (real time). Notice the sounds generated by the mole. These sounds are not rustling vegetation, but rather breaking roots as the mole forcefully pushes the soil upward.
(10.20 MB MOV)

Movie S6 This video shows earthworm escape responses to the amplified sound of a digging mole. The container filled with dirt holds 50 Diplocardia earthworms. The attached speaker is connected to a computer that is playing the recorded sound of a mole (the recordings were made with a geophone). For an example of these recordings, listen to sound file B.
(8.16 MB MOV)
